# Comparison between the diagnostic validities of Xpert MTB/RIF and interferon-γ release assays for tuberculous pericarditis using pericardial tissue

**DOI:** 10.1371/journal.pone.0188704

**Published:** 2017-12-06

**Authors:** Guocan Yu, Bo Ye, Da Chen, Fangming Zhong, Gang Chen, Jun Yang, Liliang Xu, Xudong Xu

**Affiliations:** Department of Thoracic Surgery, Hangzhou Red Cross Hospital, Hangzhou, Zhejiang, China; University of Cape Town, SOUTH AFRICA

## Abstract

**Background:**

This study aimed to assess the diagnostic performance of Xpert MTB/RIF for tuberculous pericarditis (TBP) using pericardial tissues.

**Methods:**

The study involved 30 patients admitted with suspected TBP from January–December 2016; three patients were later excluded. The interferon-γ release assay (T-SPOT.*TB*) and the Xpert MTB/RIF test were performed using peripheral blood and pericardial tissues, respectively. TBP was confirmed using pericardial histopathology and a composite reference standard (CRS). We analyzed the sensitivity, specificity, predictive value (PV), likelihood ratio (LR), and area under curve (AUC) of both assays.

**Results:**

Fourteen patients were confirmed as TBP, 10 as non-TBP, and 3 as probable TBP. The sensitivity, specificity, positive PV (PPV), negative PV (NPV), PLR, NLR, and AUC (95% confidence interval [CI]) of the Xpert MTB/RIF assay were 78.6% (49.2–95.3%) and 70.6% (44.0–89.7%); 92.3% (64.0–99.8%) and 100% (69.2–100%); 91.7% (61.5–99.8%) and 100% (73.5–100%); 80.0% (51.9–95.7%) and 66.7% (38.4–88.2%); 10.21 (1.52–68.49) and the PLR value was undefined with CRS as the reference; 0.23 (0.08–0.64) and 0.29(0.14–0.61); and 0.854 (0.666–0.959) and 0.853 (0.664–0.959), against histopathology and CRS, respectively. The sensitivity, specificity, PPV, NPV, PLR, NLR, and AUC values (95% CI) of T-SPOT.*TB* were 92.9% (66.1–99.8%) and 94.1% (71.3–99.9%); 15.4% (1.9–45.5%) and 20.0% (2.5–55.6%); 54.2% (32.8–74.5%) and 66.7% (44.7–84.4%); 66.7% (9.4–99.2%) and 66.7% (9.4–99.2%); 1.10 (0.83–1.44) and 1.18 (0.84–1.6); 0.46 (0.05–4.53) and 0.29 (0.03–2.85); and 0.541(0.340–0.733) and 0.571(0.367–0.758), against histopathology and CRS, respectively. The differences in sensitivity, PPV, and AUC of Xpert MTB/RIF and T-SPOT.*TB* were not statistically significant (P > 0.05), compared to those of histopathology and CRS. However, the differences in specificity and NPV of the two assays were significant (P < 0.05), compared to those of histopathology and CRS.

**Conclusions:**

Xpert MTB/RIF test is a valid diagnostic technique for TBP with higher sensitivity and specificity than T-SPOT.*TB*.

## Introduction

*Mycobacterium tuberculosis* (MTB) infection causes tuberculosis (TB), one of the most serious problems in public health globally [1]. The most common site of TB infection is the lung, although the bacteria can also spread to extra-pulmonary sites, leading to extrapulmonary TB (EPTB). Tuberculous pericarditis (TBP) is a type of EPTB. The incidence of TBP has been increasing along with the acquired immune deficiency syndrome (AIDS) epidemic. TBP was the most common reason for pericarditis in areas with high TB burden [2, 3]. TBP is known to increase the risk of unfavorable outcomes, including cardiac tamponade, constrictive pericarditis, and mortality [4]. The fatality rate of TBP is high (17–40%) above six months [3, 5]. Therefore, TBP should be diagnosed and treated as early as possible. However, the diagnosis of TBP is challenging and is often postponed [6], because the MTB culture and positive smear rates from pericardial effusions are very low [7]. Other tests are also used for diagnosing TBP; for instance, adenosine deaminase (ADA) is commonly used in the diagnosis of TBP. A meta-analysis showed that the overall sensitivity (95% confidence interval [CI]) of this test was 90% (86–93%) and specificity was 86% (83–89%) [8]. Interferon-γ release (T-SPOT.*TB*) test using pericardial effusion and blood can also be used for the diagnosis, and it has a sensitivity of 92% (72–99%) and 83% (62–95%) and a specificity of 92% (78–98%) and 95% (81–99%), respectively [9]. In addition, the lipoarabinomannan (LAM) assay using pericardial effusions and urinary samples has been shown to have low sensitivity, but high specificity. For instance, a previous study revealed that the sensitivity and specificity of the LAM assay using pericardial infusion were 11.6% (6.0–21.3%) and 88% (70.0–95.8%), respectively, while those using urinary samples were 17.4% (9.1–30.7%) and 93.8% (71.7–98.9%), respectively [10]. However, as pericardial effusion is difficult to obtain in several cases and these tests lack scope, the diagnosis of TBP is still very difficult.

The Xpert MTB/RIF assay shows a high accuracy for PTB and EPTB detection, and it can provide results within 2 hours [11, 12]. Previous studies have shown that this test has a high sensitivity and specificity for PTB and EPTB [13, 14]. However, the diagnostic utility of Xpert MTB/RIF on TBP has not been studied well. Only one relevant study has been published, and it showed a sensitivity of 63.8% (52.4–75.1%) and a specificity of 100% (85.6–100%) compared to those of the ADA and interferon-γ tests [15]. No studies have been conducted to evaluate the diagnostic value of Xpert MTB/RIF assay for TBP using pericardial tissues.

This study aimed to assess the diagnostic performance of the Xpert MTB/RIF test for TBP using the pericardial tissues. We also compared its performance with that of T-SPOT.*TB* assay.

## Materials and methods

### Research participants

Patients with imaging findings of pericardial effusion or pericardial thickening (CT and/or echocardiography), corresponding symptoms, pericardial effusion properties, tuberculosis contact history, or other sites of tuberculosis that were suspected of having TBP, who were admitted at Hangzhou Red Cross Hospital, the TB diagnosis and treatment center of Zhejiang province, between January 1, 2016 and December 31, 2016, were included in the study. All patients included in the study provided signed informed consent and agreed to undergo pericardial resection or pericardial fenestration to obtain the pericardial tissue and at least 9 months of follow up to assess the response to treatment. We performed pericardial fenestration through video-assisted thoracoscopic surgery (VATS) when the adhesion of pericardium was not tight. The situation of pericardial adhesion was judged by the course of disease, pericardial thickness and residual pericardial effusion. When the adhesion of pericardium was tight and constrictive pericarditis occurred, we performed pericardial resection through median sternotomy. Patients with severe underlying disease, those who were pregnant, those who showed uncertain results for T-SPOT.*TB* using peripheral blood and Xpert MTB/RIF using pericardial tissue, and those who missed the follow up were excluded from the study. Written informed consent was obtained from each patient, prior to inclusion in the study, and ethical consent was obtained from the human research ethics committee of Hangzhou Red Cross Hospital. We evaluated the medical records of all the patients studied. The diagnosis was performed based on clinical manifestations, imaging, microbiological results, pathological results, and the effect of anti-TB therapy. All patients were screened for HIV.

### Categories of diagnosis for analysis [9, 10]

After the confirmation of pericardial effusion or pericardial thickening by echocardiography or enhanced computed tomography (CT), the diagnosis of pericarditis was established. The clinical presentations of pericarditis include shortness of breath, edema, and multiple serous cavity effusions. Based on the results of microbiological culture, imaging, histopathological examination, biochemical examination of pericardial effusion, and the effect of anti-TB treatment, the patients were assigned to one of the following diagnostic groups:

1. Confirmed TB: MTB was detected in the pericardial effusion or biopsy sample by acid-fast bacilli staining, culture, polymerase chain reaction (PCR), or by the presence of granulomatous inflammations in pericardial biopsy tissue. Sputum acid-fast bacilli stain, culture, and/or by PCR showed positive results in the presence of clinical manifestations and changes in the results of imaging of TB, while other causes of pericardial effusion were excluded.
2. Highly probable TB: Clinical manifestations were observed, along with changes in the results of imaging of TB and/or increased ADA and lymphocytic predominance in pericardial effusions, with a positive response to anti-TB treatment.
3. Non TB: Microbiological cultures were negative for MTB, another diagnosis was established, or the patient improved without anti-TB treatment.

### Diagnostic specimen collection and handling

The peripheral venous blood and pericardial tissue were collected for detecting correlations. The pericardial tissues were obtained by pericardial resection or fenestration. The tissues were divided for histopathological examination and Xpert MTB/RIF assay and culture (including MTB, bacteria, and fungi), after grinding.

### Xpert MTB/RIF test

The test was performed on the pericardial tissues collected through pericardial fenestration or pericardiectomy. One milliliter of the milling pericardial tissue was placed in a pretreatment tube, and 2 mL pretreatment solution was added. The solution was agitated for 20 s and then set aside for 15 min. Next, 2 mL of the treated sample solution was added into the Xpert MTB/RIF reaction box, which was arranged in the detection module to start automatic detection. The system could automatically read the results of MTB within 2 h [12]. The reaction box and the detection modules were developed by the Cepheid company of the United States.

### T-SPOT.*TB* assay (Oxford Immunotec, Abingdon, UK)

At least 8 mL peripheral venous blood was collected from each participant, and T-SPOT.*TB* was performed by laboratory staff, who were blinded to the patients’ clinical data. The test was performed within 4 h of collection. The T-SPOT.*TB* assay has four holes: a hole (nil) to measure background interferon-producing T-cells (spot-forming cells; SFCs) as the negative control, two antigen holes (early secretary antigenic target 6-kDa protein [ESAT-6] and culture filtrate protein-10 [CFP-10]) to measure MTB-specific SFCs, and a hole to measure non-specific SFCs as the positive control (mitogen). The number of SFCs is used as the basis for classifying the results. If the spot count in the TB antigen holes exceeds a specific threshold after subtracting the number of spots in the negative control hole, non-specific SFCs are defined as positive results [16].

### Data processing and statistical analysis

The SPSS 24.0 (SPSS Inc, Chicago, IL, USA) was utilized to analyze the sensitivity, specificity, likelihood ratio (LR), predictive value (PV), and AUC of the Xpert MTB/RIF and T-SPOT.*TB* assays to evaluate their diagnostic efficiency. Histopathology and composite reference standard (CRS) were chosen as the reference diagnostic criteria. Individuals with missing data were not included in the diagnostic analysis. The mean value, standard deviation (SD), and the median and interquartile range (IQR) were used to describe the data. Chi-square (χ^2^) test and Fisher’s exact test were used for comparing the proportions. The differences between AUCs were analyzed using the STATA 14.0 (STATA Corporation, College Station, TX, USA). Differences with P < 0.05 were considered statistically significant.

## Results

**Fig 1:**
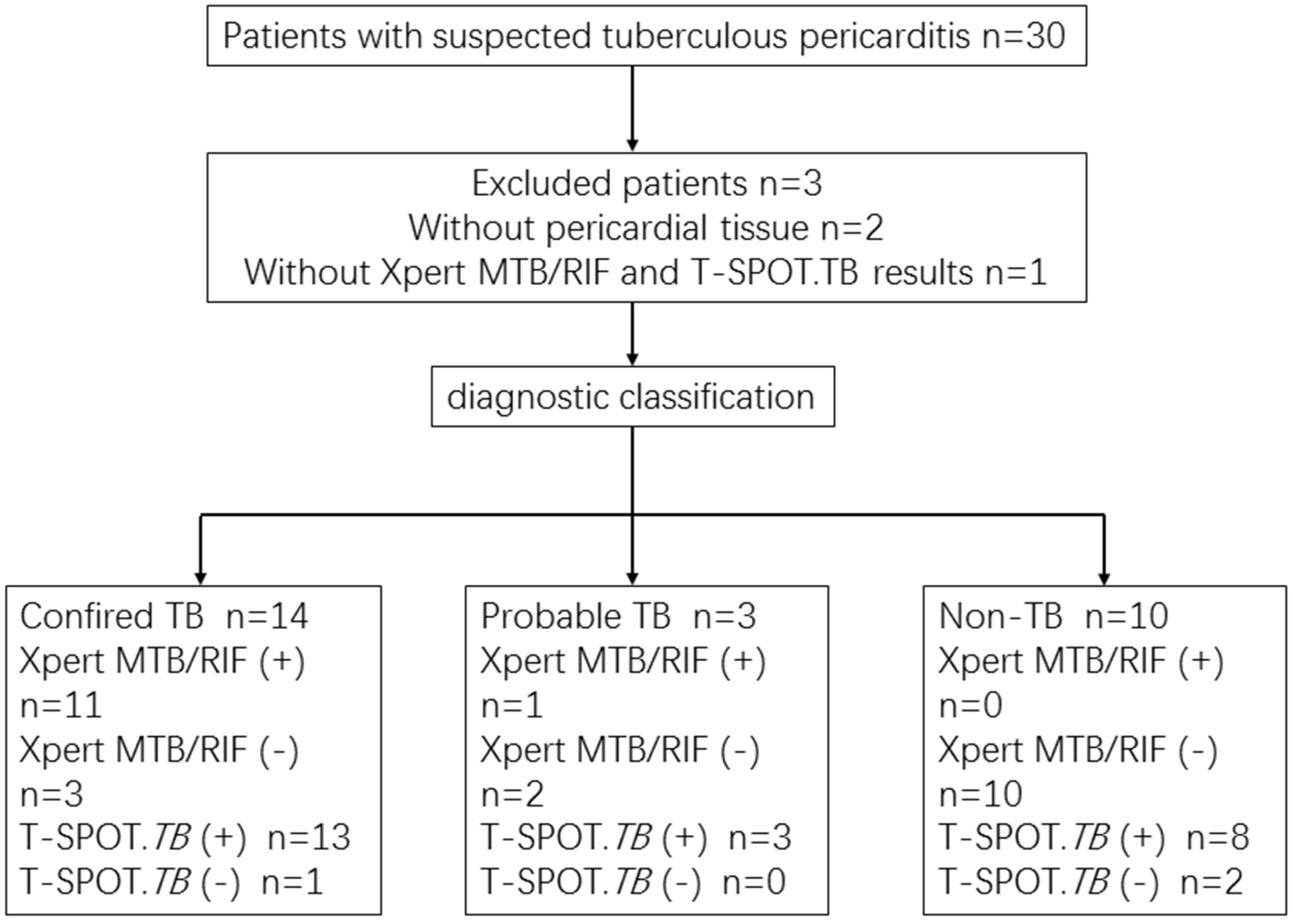
Flowchart showing the classification of patients of included in the study. TB: Tuberculosis.

Thirty patients suspected to have TBP were registered. Among them, two patients refused surgery, and one of them showed negative results in T-SPOT.*TB* and Xpert MTB/RIF assays. Thus, 27 patients were enrolled for the experiment (Fig 1).

Of the 27 patients, 14 (52%) were diagnosed as confirmed TBP (one of them was sputum smear-positive), 3 (11%) were diagnosed with highly probable TBP, and 10 (37%) had no TBP (one patient had connective tissue disease, and nine had non-specific pericarditis). Most of the patients showed no obvious pericardial effusion and could not safely undergo pericardiocentesis. Only 7 (26%) patients showed pericardial effusion after pericardial puncture. Sixteen (59%) patients showed unilateral or bilateral pleural effusion, and 9 (33%) patients showed both pleural effusion and ascites. Among the patients with confirmed TBP, 4 (29%) had PTB and 1 (17%) had both PTB and vertebral TB. Among the non-TBP patients, 3 (30%) had obsolete PTB. HIV-positive patients were not included in the study. MTB, bacterial, and fungal cultures were negative for all patients. The clinical features of patients with suspected TBP are presented in Table 1.

**Table 1:** Clinical features of patients suspected of tuberculous pericarditis.

Characteristics	All (n = 27)	Confirmed TB (n = 14)	Probable TB (n = 3)	Non-TB (n = 10)
Age year (mean ± SD)	51.0 ± 16.5	47.8 ± 19.7	42.3 ± 13.3	58.0 ± 9.5
Male (n, %)	17 (62.9)	12 (85.7)	1 (33.3)	4 (40.0)
Systolic pressure (mmHg, mean ± SD)	116 ± 18	109 ± 13	117 ± 12	126 ± 21
Diastolic pressure (mmHg, mean ± SD)	77 ± 11	75 ± 9	70 ± 11	83 ± 12
Symptoms (n, %)				
NYHA Class I–II	20 (74.1)	9 (64.2)	3 (100)	8 (80.0)
NYHA Class III–IV	7 (25.9)	5 (35.7)	0 (0)	2 (20.0)
Breathlessness	15 (55.6)	6 (42.9)	1 (33.3)	8 (80.0)
Cough	11 (40.7)	7 (50.0)	0 (0)	4 (40.0)
Edema	5 (18.5)	2 (14.3)	2 (66.7)	1 (10.0)
Fever	6 (22.2)	5 (35.7)	0 (0)	1 (10.0)
Chest tightness	21 (77.8)	11 (78.6)	1 (33.3)	9 (90.0)
Pleural effusion	25 (92.6)	14 (100)	3 (100)	8 (80.0)
Abdominal effusion	9 (33.3)	7 (50.0)	1 (33.3)	1 (10.0)
Other sites involved of TB (n, %)				
Lung	8 (29.6)	5 (35.7)	0 (0)	3 (30.0)
Vertebral tuberculosis	1 (3.7)	1 (7.1)	0 (0)	0 (0)
Laboratory examinations				
Leukocyte (*10^9^/L, Median, IQR)	5.6 (4.3–7.1)	6.3 (5.2–7.7)	5.6 (5.6–5.6)	4.4 (3.4–6.1)
Red blood cell (*10^12^/L, Median, IQR)	4.13 (3.70–4.69)	4.30 (3.89–4.87)	4.09 (3.70–)	3.90 (3.50–4.24)
ESR (n, %)	27 (100)	14 (100)	3 (100)	10 (100)
(mm/h, median, IQR)	25 (17–57)	25 (15–54)	24 (4–)	32 (18–76)
CRP (n, %)	27 (100)	14 (100)	3 (100)	10 (100)
(mg/L, median, IQR)	15 (8–34)	26 (15–36)	11 (5–)	7 (3–33)
BNP (n, %)	23 (85.2)	13 (92.9)	3 (100)	7 (70.0)
(ng/L, median, IQR)	193 (104–282)	159 (105–331)	193 (145–)	205 (34–228)
Pericardial effusion (n, %)	7 (25.9)	2 (14.3)	1 (33.3)	4 (40.0)
Echocardiographic features (n, %)	27 (100)	14 (100)	3 (100)	10 (100)
EF (%, median, IQR)	57.9 (53.3–63.6)	58.7 (54.8–63.8)	53 (52–)	57.4 (52.5–64.9)
Pericardial thickness (mm, mean ± SD)	6.9 ± 3.3	8.4 ± 2.8	8.3 ± 2.9	4.3 ± 2.3

TB: Tuberculosis, HB: Hemoglobin, ESR: Erythrocyte sedimentation rate, CRP: C reactive protein, BNP: B-type natriuretic peptide, EF: Ejection fraction.

### Evaluation of diagnostic efficiency

Histopathology and CRS are considered to be the gold standards for diagnosing TBP. CRS includes results of microbiological culture, imaging, histopathological examination, biochemical examination of pericardial effusions, and the effect of anti-TB therapy. Diagnostic accuracy of the tests was estimated and compared to that of histopathology and CRS. The results are summarized in Tables 2 and 3.

**Table 2:** Diagnostic efficiency of Xpert MTB/RIF and TSPOT.TB tests, using that of histopathology as a reference standard. P-values corresponded to statistical comparisons between Xpert MTB/RIF and TSPOT.*TB* tests.

	Sensitivity (95% CI)	Specificity (95% CI)	PPV (95% CI)	NPV (95% CI)	PLR (95% CI)	NLR (95% CI)	AUC (95% CI)
Xpert MTB/RIF	78.6% (49.2%–95.3%)	92.3% (64.0%–99.8%)	91.7% (61.5%–99.8%)	80.0% (51.9%–95.7%)	10.21 (1.52–68.49)	0.23 (0.08–0.64)	0.854 (0.666–0.959)
TSPOT.TB	92.9% (66.1%–99.8%)	15.4% (1.9%–45.5%)	54.2% (32.8%–74.5%)	66.7% (9.4%–99.2%)	1.10 (0.83–1.44)	0.46 (0.05–4.53)	0.541 (0.340–0.733)
P value	P = 0.596	P < 0.001	P = 0.031	P = 1.000	P < 0.001	P = 0.027	P = 0.001

PPV: Positive predictive value, NPV: Negative predictive value, PLR: Positive likelihood ratio, NLR: Negative likelihood ratio, AUC: Area under curve.

**Table 3:** Diagnostic value of Xpert MTB/RIF and TSPOT.*TB* tests, using that of CRS as a reference standard. P-values corresponded to statistical comparisons between Xpert MTB/RIF and TSPOT.*TB* tests.

	Sensitivity (95% CI)	Specificity (95% CI)	PPV (95% CI)	NPV (95% CI)	PLR (95% CI)	NLR (95% CI)	AUC (95% CI)
Xpert MTB/RIF	70.6% (44.0–89.7%)	100% (69.2–100%)	100% (73.5–100%)	66.7% (38.4–88.2%)	undefined	0.29 (0.14–0.61)	0.853 (0.664–0.959)
TSPOT.*TB*	94.1% (71.3–99.9%)	20.0% (2.5–55.6%)	66.7% (44.7–84.4%)	66.7% (9.4–99.2%)	1.18 (0.84–1.64)	0.29 (0.03–2.85)	0.571 (0.367–0.758)
P value	P = 0.175	P = 0.001	P = 0.033	P = 1.000	-	P = 1.000	P = 0.003

PPV: Positive predictive value, NPV: Negative predictive value, PLR: Positive likelihood ratio, NLR: Negative likelihood ratio, AUC: Area under curve.

### Xpert MTB/RIF test

Evaluation of the overall efficiency of the Xpert MTB/RIF assay revealed that it had a sensitivity, specificity, PPV, NPV, PLR, NLR, and AUC of 78.6% (49.2–95.3%), 92.3% (64.0–99.8%), 91.7% (61.5–99.8%), 80.0% (51.9–95.7%), 10.21 (1.52–68.49), 0.23 (0.08–0.64), and 0.854 (0.666–0.959), respectively, using histopathology as the reference. When CRS was used as the reference, the Xpert MTB/RIF assay had a sensitivity, specificity, PPV, NPV, PLR, NLR, and AUC of 70.6% (44.0–89.7%), 100% (69.2–100%), 100% (73.5–100%), 66.7% (38.4%–88.2%), 0.29 (0.14–0.61), and 0.853 (0.664–0.959), respectively (PLR was undefined). None of the patients included were resistant to rifampicin.

### T-SPOT.*TB* assay

Using histopathology as the reference standard, the T-SPOT.*TB* assay using peripheral blood had sensitivity, specificity, PPV, NPV, PLR, NLR, and AUC values of 92.9% (66.1–99.8%), 15.4% (1.9–45.5%), 54.2% (32.8–74.5%), 66.7% (9.4–99.2%), 1.10 (0.83–1.44), 0.46 (0.05–4.53), and 0.541 (0.340–0.733), respectively. Using CRS as the reference standard, the assay possessed sensitivity, specificity, PPV, NPV, PLR, NLR, and AUC values of 94.1% (71.3–99.9%), 20.0% (2.5–55.6%), 66.7% (44.7–84.4%), 66.7% (9.4–99.2%), 1.18 (0.84–1.64), 0.29 (0.03–2.85), and 0.571 (0.367–0.758), respectively.

### Comparison of diagnostic accuracy

The differences between the sensitivity and NPV values of the Xpert MTB/RIF assay using the pericardial tissue and the T-SPOT.*TB* assay using the peripheral venous blood, and those of histopathology and CRS, respectively, were not statistically significant (78.6% vs. 92.9%, P > 0.05; 70.6% vs. 94.1%, P > 0.05; 80% vs. 66.7%, P = 1.000; and 66.7% vs. 66.7%, P = 1.000).

The specificity and PPV of the Xpert MTB/RIF assay were higher than those of the T-SPOT.*TB* assay (92.3% vs. 15.4%, P < 0.05; 100% vs. 20%, P < 0.05; 91.7% vs. 54.2%, P < 0.05; and 100% vs. 66.7%, P < 0.05), with histopathology and CRS considered as reference standards, respectively.

When histopathology was used as the reference standard, the PLR and NLR of the Xpert MTB/RIF assay were higher than those of the T-SPOT.*TB* assay (10.21 vs. 1.10, P < 0.05 and 0.23 vs. 0.46, P < 0.05). However, there was no difference between the NLR values of Xpert MTB/RIF and T-SPOT.*TB* tests (0.26 vs. 0.26, P = 1.000) when CRS was used as the reference standard.

The AUCs from the Xpert MTB/RIF were 0.854 (P < 0.05, compared with AUC = 0.5) and 0.853 (P < 0.05, compared with AUC = 0.5), when histopathology and CRS, respectively, were used as the reference standards. The results indicated that Xpert MTB/RIF had a favorable diagnostic value for TBP. The AUCs from T-SPOT.*TB* were 0.541 (P > 0.05, compared with AUC = 0.5) and 0.571 (P > 0.05, compared with AUC = 0.5), when histopathology and CRS, respectively, were used as the reference standards, indicating that T-SPOT.*TB* was not exceptionally suitable for diagnosing TBP. The differences in the AUCs from Xpert MTB/RIF and T-SPOT.*TB* were statistically significant (0.854 vs. 0.541, P < 0.05 and 0.853 vs. 0.571, P < 0.05), with histopathology and CRS used as the reference standards, respectively. The AUCs of Xpert MTB/RIF were obviously higher than those of T-SPOT.*TB*.

## Discussion

The Xpert MTB/RIF assay is a rapid, automatic, and WHO-approved TB diagnostic test, which can not only diagnose MTB complex DNA, but also confirm rifampicin resistance caused by the rpoB gene mutations [11]. This assay is also the preferred diagnostic method for detecting drug-resistant or HIV-associated TB [17]. It is widely used for diagnosing PTB and EPTB. Several studies have reported the diagnostic efficiency of Xpert MTB/RIF for PTB and EPTB. For instance, Chew MY et al. showed that Xpert MTB/RIF had a sensitivity of 75% and a specificity of 99.5% in an intermediate-burden setting for diagnosing PTB [18]. A study in Ethiopia showed that Xpert MTB/RIF had a sensitivity of 93.5% (78.3–98.9%) and a specificity of 69.2% (66.4–70%) for tuberculous lymphadenitis [19]. In case of pleural TB, Xpert MTB/RIF showed a sensitivity of 22.5% (12.4–37.6%) and a specificity of 98% (89.2–99.7%) [20].

T-SPOT.*TB* is another frequently used technique for diagnosing TB in various systems [21–23]. The test can be performed on blood and other body fluids [9, 22] (such as pleural effusion and pericardial effusion). However, in our hospital, all samples other than blood are tested by the T-SPOT.*TB* assay. Therefore, this assay was not used on pericardial effusion or tissues.

TBP is a type of EPTB, which accounts for about 4% of pericarditis cases in the developed countries [3]; in South Africa, this rate is as high as 60% [24]. In this study, TB was found to be the cause of pericarditis in 63% (17/27) of the patients, which is a rate higher than that reported previously [25]. This might be because our hospital is a diagnosis and treatment center for TB in the Zhejiang province, with a naturally high proportion of patients with TB. We consider that an imperfect reference standard may lead to misclassification of samples in diagnostic validity studies. In case of EPTB, histopathology is an imperfect reference standard. This reference standard would lead to an underestimation of the true specificity of Xpert MTB/RIF. CRS is a composite standard, which includes results of several tests or clinical conditions that may sometimes be reclassified as false positive results of Xpert MTB/RIF as true positive results, and thus, lead to an increase in Xpert MTB/RIF specificity. However, CRS itself may have reduced specificity that could result in apparent false-negative Xpert MTB/RIF results, leading to an underestimation of the true sensitivity of Xpert MTB/RIF. Therefore, a comparative study with the two reference standards, histopathology and CRS, might provide a more credible range for sensitivity and specificity.

There have been very few reports on the diagnostic utility of Xpert MTB/RIF for TBP. The Xpert MTB/RIF assay was performed on pericardial effusions in previously published studies [15, 26]. The utility of Xpert MTB/RIF using pericardial tissues has never been reported. In this study, the vast majority of patients (20/27) did not show adequate pericardial effusion at the time of admission to safely perform pericardial puncture. Therefore, we assessed the diagnostic value of Xpert MTB/RIF on pericardial tissues, which were obtained by pericardial fenestration or resection. To the best of our knowledge, this is the first report to assess the diagnostic utility of Xpert MTB/RIF using pericardial tissue for TBP and compare it with T-SPOT.*TB* test.

The T-SPOT.*TB* assay was found to be more sensitive than the Xpert MTB/RIF assay, although the difference was not statistically significant, regardless of the reference standard used. Xpert MTB/RIF assay had higher specificity (92.3% and 100%, with histopathology and CRS as the reference standards, respectively) and PPV (91.7% and 100%) using pericardial tissue than those of the T-SPOT.*TB* assay. This result was consistent with the result of a previous study (specificity and PPV were both 100%) [15], in which Xpert MTB/RIF was performed using pericardial effusion. However, it is still unknown whether the diagnostic value of Xpert MTB/RIF using pericardial effusion and pericardial tissue is different. Further studies would be needed to confirm this hypothesis.

In contrast, the specificity of the T-SPOT.*TB* assay using the peripheral blood was very low (15.4% and 20.0%, respectively, with histopathology and CRS as the reference). These values were significantly lower than the previously reported ones [9]. The PPV of T-SPOT.*TB* assay, compared to histopathology and CRS, was also relatively low. This might be because China has a high incidence of TB. Nearly half the population has been reported to be infected with MTB [27], and patients with latent infection may show positive results.

The specificity and PPV of Xpert MTB/RIF and T-SPOT.*TB* assays were increased when CRS was used as the reference. Although histopathology is considered the gold standard for diagnosing TBP, it is not a perfect reference method. The results of pathological diagnosis are closely related to the location of the biopsy site, the quality of equipment, and the experience of the physician. CRS classifies TB based on positive results from one of the several criteria, including culture, clinical manifestation, histopathology, imaging, and response to treatment, resulting in the reclassification of false positives to true positives and increased specificity and PPV [28].

Receiver operating curve (ROC) analysis showed that the AUC of Xpert MTB/RIF was significantly higher than that of T-SPOT.*TB*, regardless of the reference standard. The T-SPOT.*TB* assay was found to be not important for diagnosing TBP, while the Xpert MTB/RIF test played a crucial role in it.

There were a number of limitations of the present study. The number of patients studied was limited and included no positive cases of MTB culture. This might have caused a bias in the evaluation of the effectiveness of the diagnosis. Furthermore, this was a single-center retrospective study, performed in the TB diagnosis and treatment center of the Zhejiang province. This might have led to a selection bias, as the patients enrolled would always be suspected to have TB. This might also explain the high prevalence rate of TBP in this study, compared to the previous ones [9, 25].

Studies on the viability of the Xpert MTB/RIF assay using pericardial tissues for the diagnosis of TBP are rare; our study may have some clinical significance, although the applicability of an invasive operation may be limited. In future studies, multi-center cooperation is needed to assess the diagnostic validity of Xpert MTB/RIF in a wider population.

## Conclusions

In summary, the diagnostic validity of Xpert MTB/RIF using pericardial tissue for TBP has not been studied before, and to the best of our knowledge, this was the first such report. We concluded that the Xpert MTB/RIF assay using pericardial tissue showed high specificity and PPV for diagnosing TBP. The specificity and PPV of T-SPOT.*TB* were low, but the sensitivity was high. The Xpert MTB/RIF assay had better diagnostic efficiency for TBP than T-SPOT.*TB* assay had. Thus, Xpert MTB/RIF is a suitable diagnostic method for TBP, and the diagnostic value of T-SPOT.*TB* is not exceptional.

## Supporting information

S1 Supporting Information Data generated or analyzed in this study, including patient characteristics, raw data, and funding. (ZIP)
