# Supplementary material for: Comparison between the diagnostic validities of Xpert MTB/RIF and interferon-γ release assays for tuberculous pericarditis using pericardial tissue
Source: PLoS One. 2017 Dec 6;12(12):e0188704. doi: 10.1371/journal.pone.0188704 (PMC5718425; doi:10.1371/journal.pone.0188704)
Supplement: S1 Supporting Information — (ZIP) [file pone.0188704.s001.zip › S1_Supporting_Information/S1 Table.docx]

**Clinical features of patients suspected of tuberculous pericarditis.**

| Characteristics | All (n = 27) | Confirmed TB (n = 14) | Probable TB (n = 3) | Non-TB (n = 10) |
| --- | --- | --- | --- | --- |
| Age year (mean ± SD) | 51.0±16.5 | 47.8±19.7 | 42.3±13.3 | 58.0±9.5 |
| Male (n, %) | 17 (62.9) | 12 (85.7) | 1 (33.3) | 4 (40.0) |
| Systolic pressure (mmHg, mean±SD) | 116±18 | 109±13 | 117±12 | 126±21 |
| Diastolic pressure (mmHg, mean±SD) | 77±11 | 75±9 | 70±11 | 83±12 |
| Symptoms (n, %) |  |  |  |  |
| NYHA Class I–II | 20 (74.1) | 9 (64.2) | 3 (100) | 8 (80.0) |
| NYHA Class III–IV | 7 (25.9) | 5 (35.7) | 0 (0) | 2 (20.0) |
| Breathlessness | 15 (55.6) | 6 (42.9) | 1 (33.3) | 8 (80.0) |
| Cough | 11 (40.7) | 7 (50.0) | 0 (0) | 4 (40.0) |
| Edema | 5 (18.5) | 2 (14.3) | 2 (66.7) | 1 (10.0) |
| Fever | 6 (22.2) | 5 (35.7) | 0 (0) | 1 (10.0) |
| Chest tightness | 21 (77.8) | 11 (78.6) | 1 (33.3) | 9 (90.0) |
| Pleural effusion | 25 (92.6) | 14 (100) | 3 (100) | 8 (80.0) |
| Abdominal effusion | 9 (33.3) | 7 (50.0) | 1 (33.3) | 1 (10.0) |
| Concurrent disease (n, %) |  |  |  |  |
| HIV infection | 0 (0) | 0 (0) | 0 (0) | 0 (0) |
| Type 2 diabetesmellitus | 1 (3.7) | 1 (7.1) | 0 (0) | 0 (0) |
| Malignant tumor | 1 (3.7) | 1 (7.1) | 0 (0) | 0 (0) |
| Chronic kidney disease | 1 (3.7) | 1 (7.1) | 0 (0) | 0 (0) |
| Hypothyroidism | 2 (7.4) | 0 (0) | 1 (33.3) | 1 (10.0) |
| Syphilis | 2 (7.4) | 1 (7.1) | 0 (0) | 1 (10.0) |
| Arrhythmia | 2 (7.4) | 0 (0) | 1 (33.3) | 1 (10.0) |
| Hypertension | 5 (18.5) | 1 (7.1) | 0 (0) | 4 (40.0) |
| Pneumoconiosis | 1 (3.7) | 1 (7.1) | 0 (0) | 0 (0) |
| Other sites involved of TB (n, %) |  |  |  |  |
| Lung | 8 (29.6) | 5 (35.7) | 0 (0) | 3 (30.0) |
| Vertebral tuberculosis | 1 (3.7) | 1 (7.1) | 0 (0) | 0 (0) |
| Laboratory examinations |  |  |  |  |
| Leukocyte (*10^9^/L, Median, IQR) | 5.6 (4.3-7.1) | 6.3 (5.2-7.7) | 5.6 (5.6-5.6) | 4.4 (3.4-6.1) |
| Red blood cell (*10^12^/L, Median, IQR) | 4.13 (3.70-4.69) | 4.30 (3.89-4.87) | 4.09 (3.70- ) | 3.90 (3.50-4.24) |
| HB (Median, IQR) | 118 (105-130) | 120 (110-137) | 114 (100- ) | 112 (98-132) |
| ESR (n, %) | 27 (100) | 14 (100) | 3 (100) | 10 (100) |
| (mm/h, median, IQR) | 25 (17-57) | 25 (15-54) | 24 (4- ) | 32 (18-76) |
| CRP (n, %) | 27 (100) | 14 (100) | 3 (100) | 10 (100) |
| (mg/L, median, IQR) | 15 (8-34) | 26 (15-36) | 11 (5- ) | 7 (3-33) |
| BNP (n, %) | 23 (85.2) | 13 (92.9) | 3 (100) | 7 (70.0) |
| (ng/L, median, IQR) | 193 (104-282) | 159 (105-331) | 193 (145- ) | 205 (34-228) |
| Pericardial effusion (n, %) | 7 (25.9) | 2 (14.3) | 1 (33.3) | 4 (40.0) |
| Appearance (n, %) | 7 (25.9) | 2 (14.3) | 1 (33.3) | 4 (40.0) |
| Yellow and clear | 5 (18.5) | 2 (14.3) | 1 (33.3) | 2 (20.0) |
| Bloody | 2 (7.4) | 0 (0) | 0 (0) | 2 (20.0) |
| Total protein (n, %) | 7 (25.9) | 2 (14.3) | 1 (33.3) | 4 (40.0) |
| (g/L, median, IQR) | 52.9 (43.9-58.8) | 44.8 (32.7- ) | 43.9 (43.9-43.9) | 55.9 (51.0-64.4) |
| ADA (n, %) | 7 (25.9) | 2 (14.3) | 1 (33.3) | 4 (40.0) |
| (U/L, median, IQR) | 13 (12-30) | 53 (30- ) | 6 (6-6) | 12.5 (12.0-15.3) |
| LDH (n, %) | 7 (25.9) | 2 (14.3) | 1 (33.3) | 4 (40.0) |
| (U/L, median, IQR) | 354 (174-919) | 981 (613- ) | 135 (135-135) | 327 (205-778) |
| Echocardiographic features (n, %) | 27 (100) | 14 (100) | 3 (100) | 10 (100) |
| EF (%, median, IQR) | 57.9 (53.3-63.6) | 58.7 (54.8-63.8) | 53 (52- ) | 57.4 (52.5-64.9) |
| Pericardial thickness(mm, mean±SD) | 6.9±3.3 | 8.4±2.8 | 8.3±2.9 | 4.3±2.3 |

TB: Tuberculosis, HB: Hemoglobin, ESR: Erythrocyte sedimentation rate, CRP: C reactive protein, BNP: B-type natriuretic peptide, ADA: Adenosine deaminase, LDH: lactate dehydrogenase, EF: Ejection fraction.
