# Supplementary figures and images for: Comparison between the diagnostic validities of Xpert MTB/RIF and interferon-γ release assays for tuberculous pericarditis using pericardial tissue
Source: PLoS One. 2017 Dec 6;12(12):e0188704. doi: 10.1371/journal.pone.0188704 (PMC5718425; doi:10.1371/journal.pone.0188704)

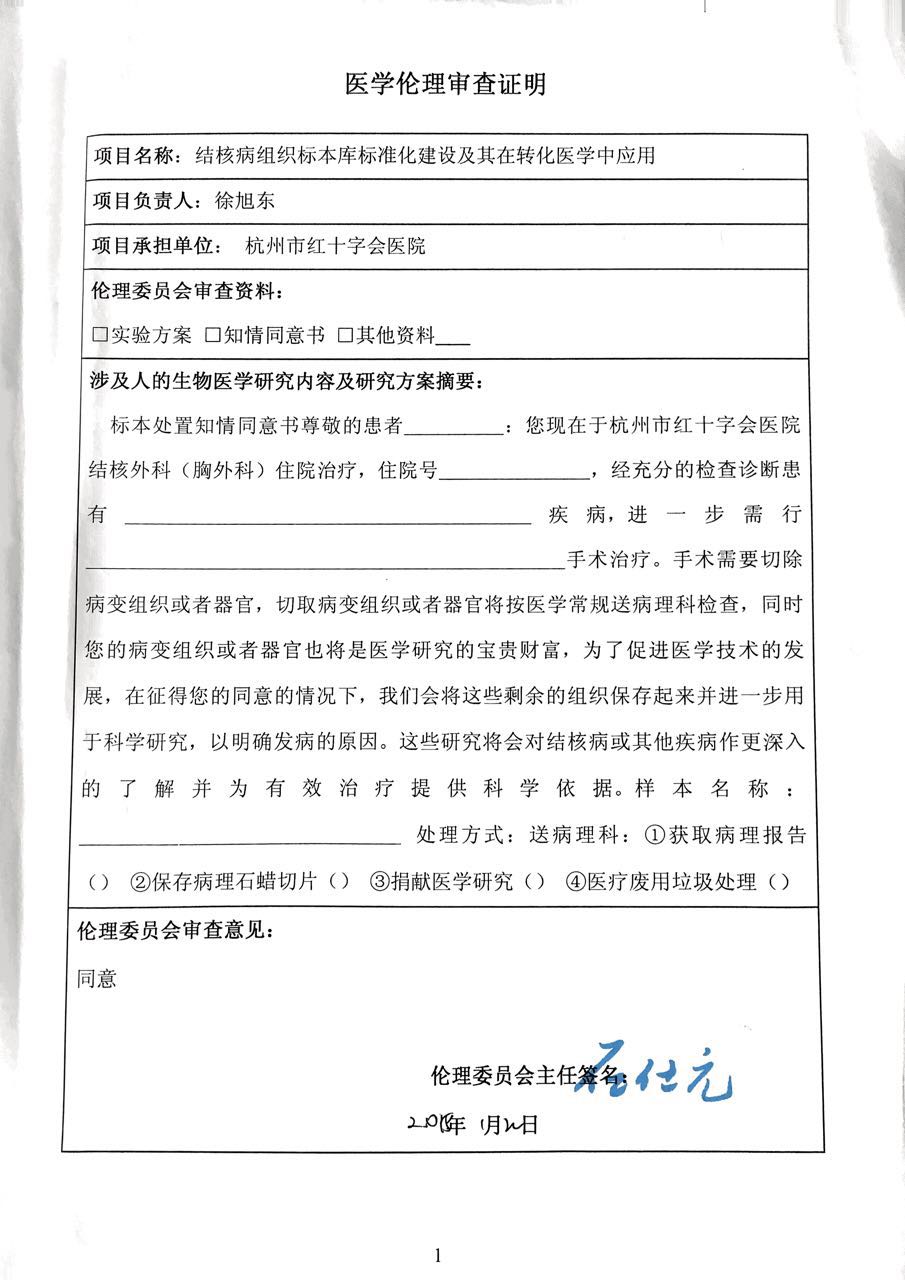

Supplement: S1 Supporting Information — (ZIP) [file pone.0188704.s001.zip › S1_Supporting_Information/ethic and funding/ethic.jpg]

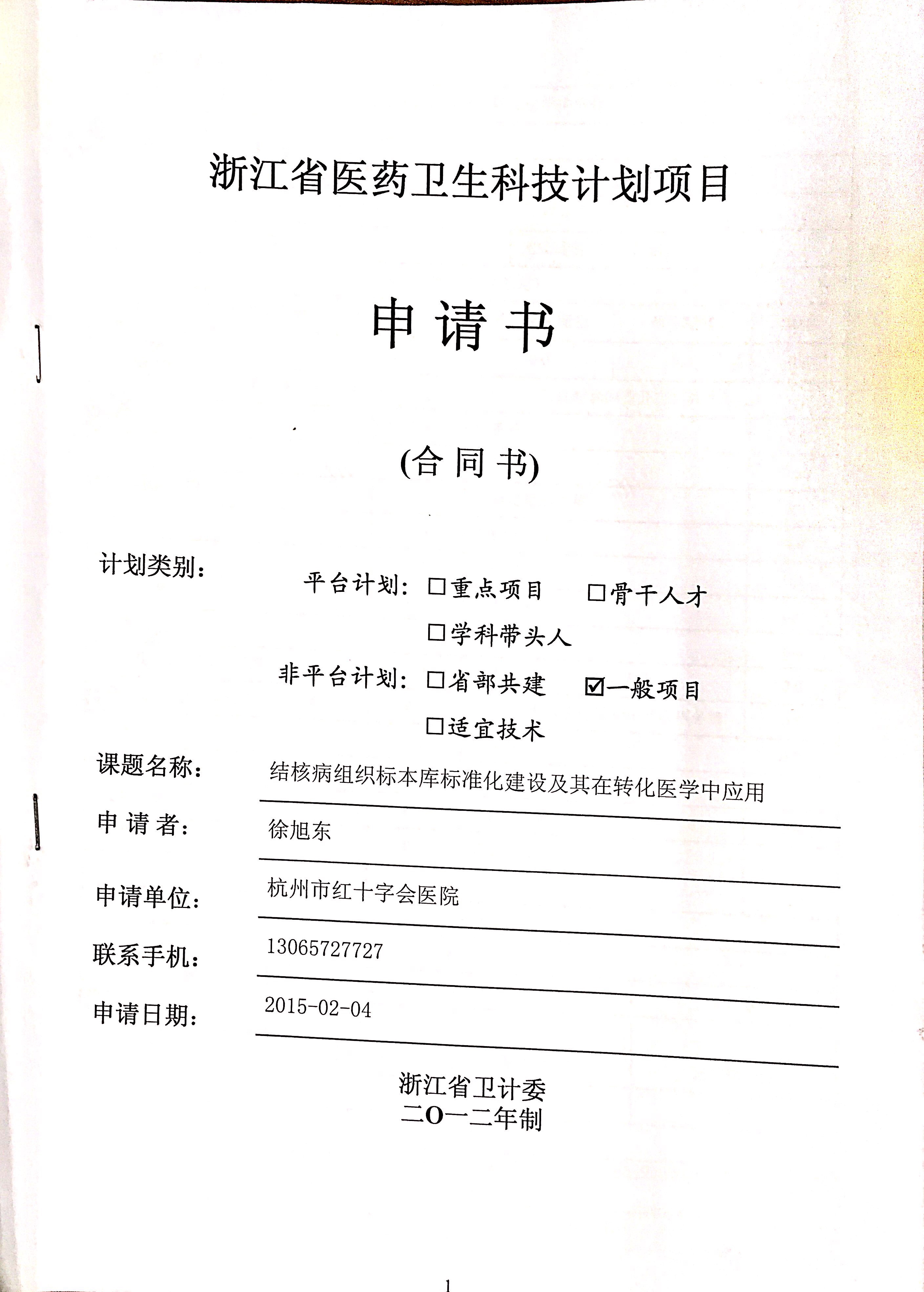

Supplement: S1 Supporting Information — (ZIP) [file pone.0188704.s001.zip › S1_Supporting_Information/ethic and funding/funding1.jpg]

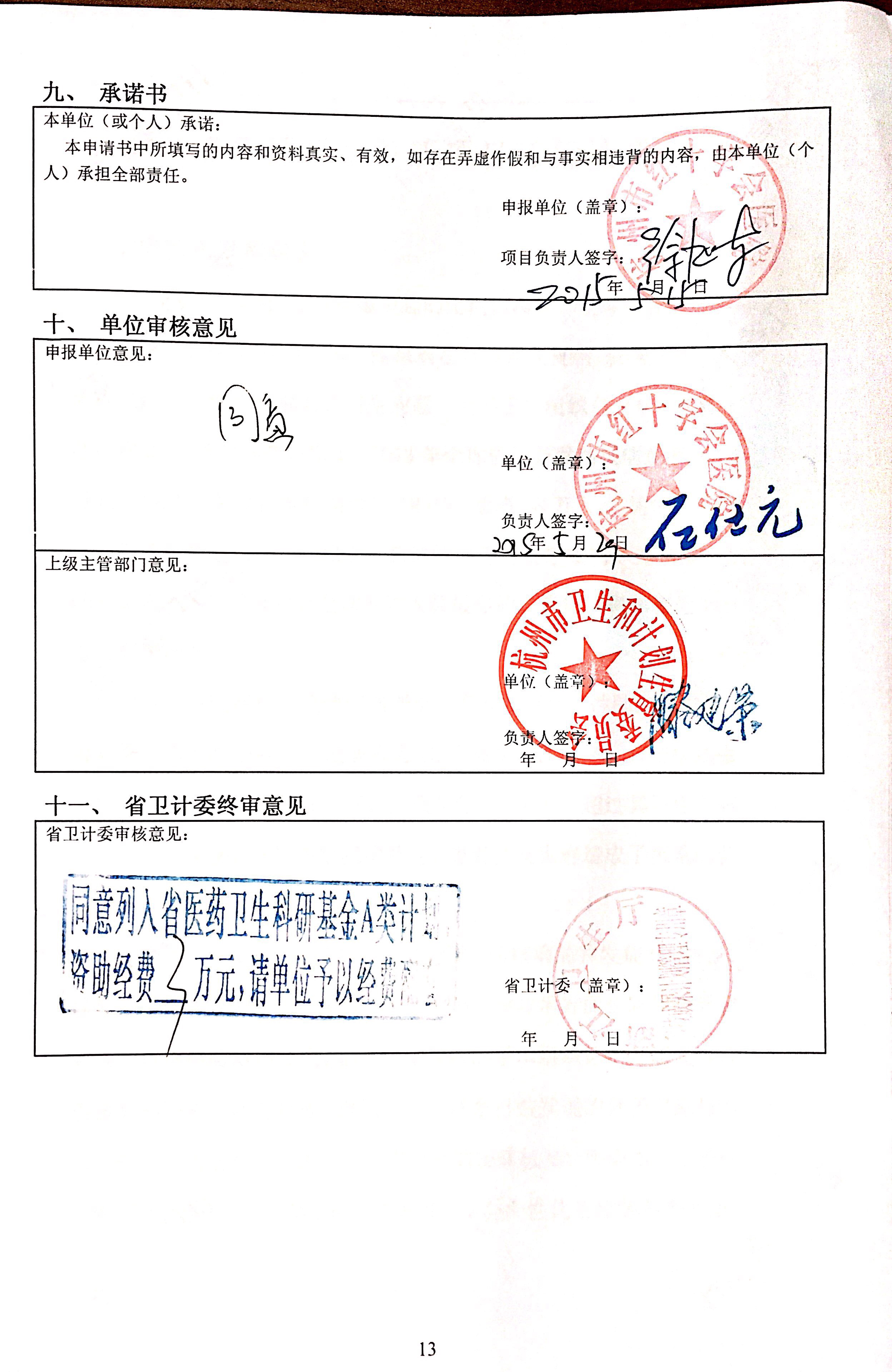

Supplement: S1 Supporting Information — (ZIP) [file pone.0188704.s001.zip › S1_Supporting_Information/ethic and funding/funding2.jpg]
